# Knowledge of and acceptability towards human papilloma virus vaccine in Saudi Arabia: A cross-sectional survey study

**DOI:** 10.1097/MD.0000000000041941

**Published:** 2025-03-28

**Authors:** Mohammed Samannodi, Shahad Mohammed Mousa, Lama Adnan Mandourah, Shayma Saleh Al-Rubaki, Maram Sulaiman Gubari, Wala Salem Fallatah, Sarah Tayeb, Mohammed A. Almatrafi, Mohammed Shaikhomer, Hassan Alwafi, Ahmad A. Imam, Enad Alsolami

**Affiliations:** a Department of Medicine, College of Medicine, Umm Al-Qura University, Makkah, Saudi Arabia; b Department of Medicine, King Fahad Armed Forces Hospital, Ministry of Defense, Jeddah, Saudi Arabia; c Department of Pediatrics, Soliman Fakeeh Hospital, Jeddah, Saudi Arabia; d General Medicine, Batterjee Medical College, Jeddah, Saudi Arabia; e General Medicine Practice, Batterjee Medical College, Jeddah, Saudi Arabia; f Al Nuayriah General Hospital, Ministry of Health, Al Nuayriah, Saudi Arabia; g Division of Adult Infectious Diseases, Department of Medicine, King Fahad Armed Forces Hospital, Ministry of Defense Health Services, Jeddah, Saudi Arabia; h Department of Pediatrics, College of Medicine, Umm Al-Qura University, Saudi Arabia, Makkah; i Department of Internal Medicine, Faculty of Medicine, King Abdulaziz University, Jeddah, Saudi Arabia; j Department of Pharmacology and Toxicology, College of Medicine, Umm Al-Qura University, Saudi Arabia; k Department of Internal Medicine, College of Medicine, University of Jeddah, Jeddah, Saudi Arabia.

**Keywords:** acceptability, human papilloma virus, knowledge, Saudi Arabia, vaccine

## Abstract

Vaccination against human papilloma virus (HPV) play a major role in preventing infection with HPV among heterosexual couples. The aim of this study was to assess public knowledge and attitude towards HPV vaccine in Saudi Arabia. This is an online cross-sectional survey study that was conducted between May and June 2023. This study utilized the convenience sampling technique to recruit the study participants. The study participants were invited to participate in this study through social media platforms (X, Facebook, and WhatsApp). Multiple logistic regression was performed to assess the factors associated with better knowledge level and the findings were presented as odds ratios (OR) with 95% confidence intervals (CI) and corresponding p-values. A total of 819 participants were included in the analysis. A total of 355 participants (43.3%) had a good knowledge score and 464 participants (56.7%) had poor knowledge. The total mean of knowledge score was (3.22 ± 2.44). As the table shown, single participants reported a significant higher knowledge score mean (3.59 ± 2.52) compared to married (2.84 ± 2.32) (*P* = .0001). Participants aged between 18 to 29 years reported a significant higher knowledge score mean (3.57 ± 2.52) compared to participants aged between 40 and 49 years (2.60 ± 2.21) (*P* = .0001). Participants who lived in Eastern area had significantly higher odds of knowledge compared to other areas (OR = 2.19, 95% CI = 1.45 − 3.33, *P* = .001). Participants who worked in medical field had significantly higher odds of having good knowledge compared to other jobs (OR = 3.65, 95% CI = 2.39–5.57, *P* = .0001). Participants who had 2 sexual partners have you had in the past 2 years had a significant higher odd of having good knowledge (OR = 2.05, 95% CI = 1.02–4.12, *P* = .04). This study identified that a considerable proportion of the study participants demonstrated poor level of knowledge of HPV vaccine. Participants who lived in Eastern area, those who worked in medical field, and those who had 2 sexual partners have you had in the past 2 years had a significant higher odd of having good knowledge. Future studies should be directed towards developing educational campaign to improve public awareness of HPV.

## 1. Introduction

Human papillomavirus (HPV) is a virus that infects various body tissues, especially the skin, lungs, vulva, pharynx, throat, tonsils, back of the tongue, cervix, anus, vagina, and penis.^[1]^ Human papillomavirus plays a pivotal role in head and neck cancer, primarily oral and oropharyngeal squamous cell carcinoma, and is 1 of the most common sexually transmitted infections.^[2]^ Various factors increase the risk of HPV infection, and it is not clear why HPV infection tends to occur in specific areas of the genital tract.^[3]^

Proper clinical management can avoid the development of viral tumors of this disease and dental care is the first line of clinical evaluation of this virus in the mouth.^[4]^ There is a wide range of human papillomavirus genes in the oral mucosa, and HPV with low-risk genotypes has a low role in the carcinogenesis of oral squamous cell carcinoma. Vaccination against HPV types 6, 11, 16, and 18 are expected to reduce the risk of HPV 16 carcinogenesis in the nasopharynx and oral cavity and reduce the prevalence of infection.^[5]^ Human papillomavirus differs from other viruses that infect the oral epithelium by causing proliferative changes in these cells, resulting in the appearance of benign and malignant tumors. HPV 2 and 4 cause common cutaneous warts (vulgar warts), while HPV 6 and 11 cause genital warts (vulgar warts) and common oral solitary papilloma.^[6]^

Considering social factors in HPV awareness campaigns to increase HPV vaccination has a major role, as there was a difference in the level of awareness among people about HPV across social factors. The people who have a lower level of education have a lower level of awareness.^[7]^ Human papillomavirus causes cervical cancer, disproportionately affects low-income and minority women. It is urgent that not only improve HPV vaccination rates overall but also focus on at-risk populations to prevent further lack of correspondence in cervical cancer incidence.^[8]^

However, medical caregivers’ failure to discuss vaccination with their patients is an important factor in non-vaccination, so future research should focus on improving both HPV vaccination initiation and completion in low-income populations.^[9]^ Male circumcision, vaccination against this virus, smoking cessation, and using condoms play a major role in preventing infection with HPV among heterosexual couples.^[10]^ There are compensatory campaigns that have proven their effectiveness in reducing the incidence of HPV, so preventive vaccination was targeted at children before they start having sex.^[11]^ The aim of this study was to assess public knowledge and attitude towards HPV vaccine in Saudi Arabia.

## 2. Methods

### 2.1. Study design

This is an online cross-sectional survey study that was conducted between May and June 2023.

### 2.2. Study population and sampling technique

The study population for this research comprised of the general public in Saudi Arabia. The inclusion criteria for this research were male or female adults, aged 18 years and older, who are currently living in Saudi Arabia. Participants who are diagnosed with HPV were excluded from this study. Besides, other exclusion criteria included not providing consent to participate.

This study utilized the convenience sampling technique to recruit the study participants. The study participants were invited to participate in this study through social media platforms (X, Facebook, and WhatsApp). The study inclusion criteria were mention in the title page of the initiation letter. The study participants were informed that personal information will not be shared. Besides, they were informed that the study findings will be presented anonymized.

### 2.3. Study tool

The questionnaire tool for this study was formed based on extensive literature review.^[12,13]^ The questionnaire tool comprised of 3 sections. The first section asked the study participants about their demographic characteristics (marital status, age, living area, education level, occupation, comorbidities, and how many sexual partners have they had in the past 2 years). The second section examined participants knowledge of HPV and asked the participants about HPV cause, prevention, and vaccination. The third section examined participants’ attitude towards HPV vaccination.

### 2.4. Questionnaire piloting

The questionnaire tool was revised by 2 experts’ infectious diseases specialists to assess its validity and reliability. This was followed by small pilot study on 30 participants who meet the inclusion criteria in order to assess the understandability of the questionnaire tool.

### 2.5. Sample size

Using a statistical power of 80.0%, margin of error of 5%, and a confidence interval of 95%, the required sample size was deemed to be at least 385 participants.

### 2.6. Ethical approval

Ethical approval for this research was obtained from the Biomedical Research Ethics Committee in Umm Al-Qura University, Approval No. (HAPO-02-K-012-2023-02-1447).

### 2.7. Statistical analysis

Statistical methods were used to analyze the data, including the calculation of descriptive statistics such as the frequency and percentage for categorical variables, the mean, and the standard deviation for the continuous variables. The analysis of variance (ANOVA) test and the independent t-test were performed when applicable. Tukey post hoc test was conducted for multiple group comparisons. Multiple logistic regression was performed to assess the factors associated with better knowledge level and the findings were presented as odds ratios with 95% confidence intervals (CI) and corresponding p-values. The level of significance was defined as α = 0.05. All calculations and analyses were carried out with the Statistical Package of Social Sciences (SPSS) software, version 29.

## 3. Results

A total of 819 participants were included in the analysis. Regarding marital status, the majority were single (n = 410, 50.1%), followed by married individuals (n = 383, 46.8%). The most common age group was 18 to 29 years, accounting for 467 participants (57.0%). In terms of living area, the west region was the most represented, with 239 participants residing there (29.2%). The majority of the participants had a university degree (n = 612, 74.7%), while primary and middle school education levels were less common (n = 12, 1.5%) and (n = 15, 1.8%), respectively. Most participants had no comorbidities (n = 730, 89.1%). Additional details about demographic characteristics of the participants are provided in Table 1.

**Table 1:** The demographic characteristics of the participants.

Demographic variable		Frequency	Percentage
Marital status	Single	410	50.1
	Married	383	46.8
	Divorced	18	2.2
	Widowed	8	1.0
Age (yr)	18 to 29	467	57.0
	30 to 39	152	18.6
	40 to 49	130	15.9
	50 to 59	62	7.6
	60 yr and older	8	1.0
Living area	West	239	29.2
	East	202	24.7
	North	44	5.4
	South	203	24.8
	Middle	131	16.0
Education level	Primary school	12	1.5
	Middle school	15	1.8
	High school	180	22.0
	University level	612	74.7
Occupation	Not working	376	45.9
	Medical field	172	21.0
	Other	271	33.1
Comorbidities	No	730	89.1
	Yes	89	10.9
How many sexual partners have you had in the past 2 yr?	0	457	55.8
	1	273	33.3
	2	46	5.6
	3 or more	43	5.3

The knowledge items regarding HPV are presented in Table 2. Regarding the transmission of HPV, a total of 452 participants correctly identified sexual contact as main cause (34.6%), while a significant proportion (55.2%) reported uncertainly. Most of participants (62.1%) identified vaccination, early screening, and handwashing as the most effective methods to prevent infection. However, (n = 229, 27.9%) expressed a lack of knowledge. In terms of vaccination, 38.6% of participants confirmed its available in Saudi Arabia. Regarding the recommended age for receiving the HPV vaccine, 37.7% correctly identified the 11 to 27 age range, but 53.8% were unaware. When asked whether the vaccine protects against all types of HPV, only 275 participants (33.6%) correctly answered, while the majority (58.9%) were uncertain. Additional details about the knowledge of HPV are provided in Table 2.

**Table 2:** The answers for HPV knowledge items.

		Frequency	Percentage
Is sexual contact a main cause of HPV infection transmission?	Yes*	283	34.6
	No	84	10.3
	I don’t know	452	55.2
Does the annual Pap smear reduce the risk of cervical cancer?	Yes*	391	47.7
	No	63	7.7
	I don’t know	365	44.6
What is the best way to prevent HPV infection? ^#^	The vaccine against the virus, early screening, and handwashing*	509	62.1
	Using public restrooms	28	3.4
	Hand washing	65	7.9
	I don’t know	229	27.9
Is the HPV vaccine available in hospitals and clinics in Saudi Arabia?	Yes*	316	38.6
	No	33	4.0
	I don’t know	470	57.4
What is the recommended age for receiving the HPV vaccine in Saudi Arabia?	11 to 27*	309	37.7
	1 to 10	25	3.1
	More than 50	44	5.4
	I don’t know	441	53.8
Does the HPV vaccine protect against all types of the virus	No*	275	33.6
	I Don’t know	482	58.9
	Yes	62	7.6
Does the vaccine itself cause HPV infection?	No*	340	41.5
	I Don’t know	439	53.6
	Yes	40	4.9
Does the HPV vaccine prevent the occurrence of genital warts, cervical cancer, and penile cancer?	Yes*	215	26.3
	No	84	10.3
	I don’t know`	520	63.5

HPV = human papilloma virus.

* Correct answer.

A total of 355 participants (43.3%) had a good knowledge score and 464 participants (56.7%) had poor knowledge. A total of 620 participants consulted a doctor if they notice warts in the genital area (75.7%), and (n = 645, 78.8%) advised their partner to receive the HPV vaccine. Most of participants (82.8%) advised those at high risk of contracting HPV to receive the vaccine. However, (n = 669, 81.7%) advise women to have an annual Pap smear. Regarding the desire to receive the HPV vaccine, the majority (n = 473,37.7%) have the desire, and 67 participants (8.2%) prevented to be vaccinated because they feel that they are not at risk. Additional details about the HPV vaccine are provided in Table 3.

**Table 3:** Participants’ attitude toward the HPV vaccine.

Attitude item		Frequency	Percentage
Do you consult a doctor if you notice warts in the genital area?	No	199	24.3
	Yes	620	75.7
Would you advise your partner to receive the HPV vaccine?	No	174	21.2
	Yes	645	78.8
Would you advise those at high risk of contracting HPV to receive the vaccine?	No	141	17.2
	Yes	678	82.8
Would you advise women to have an annual Pap smear?	No	150	18.3
	Yes	669	81.7
Do you have the desire to receive the HPV vaccine?	No	346	42.2
	Yes	473	57.8

HPV = human papilloma virus.

The total mean of knowledge score was (3.22 ± 2.44). As the table shown, single participants reported a significant higher knowledge score mean (3.59 ± 2.52) compared to married (2.84 ± 2.32) (*P* = .0001). Participants aged between 18 to 29 years reported a significant higher knowledge score mean (3.57 ± 2.52) compared to participants aged between 40 and 49 years (2.60 ± 2.21) (*P* = .0001). Additional details about knowledge score stratified by the demographics are provided in Table 4.

**Table 4:** The knowledge score stratified by the demographic characteristics.

		Knowledge score		*P*-value
		Mean	SD	
Marital status	Single	3.59	2.52	<.001
	Married	2.84	2.32	
	Divorced	3.11	2.27	
	Widowed	3.00	2.33	
Age	18 to 29	3.57	2.52	<.001
	30 to 39	2.92	2.24	
	40 to 49	2.60	2.21	
	50 to 59	2.66	2.36	
	60-	2.75	2.38	
Living area	West	3.12	2.51	.40
	East	3.86	2.41	
	North	3.23	2.43	
	South	2.76	2.30	
	Middle	3.13	2.42	
Education level	Primary	3.92	2.84	.22
	Middle	2.00	1.56	
	High	2.78	2.29	
	University	3.37	2.47	
Occupation	Not working	2.91	2.34	.72
	Medical	4.81	2.26	
	Other	2.65	2.28	
Comorbidities	No	3.27	2.45	.12
	Yes	2.84	2.31	
How many sexual partners have you had in the past 2 yr?	0	3.32	2.48	.46
	1	3.04	2.42	
	2	3.28	2.20	
	3 or more	3.23	2.41	

SD = standard deviation.

A multiple logistic regression model was obtained to assess the factors affected the knowledge level. Participants who lived in Eastern area had significantly higher odds of knowledge compared to other areas (OR = 2.19, 95% CI = 1.45 − 3.33, *P* = .001). Participants who worked in medical field had significantly higher odds of having good knowledge compared to other jobs (OR = 3.65, 95% CI = 2.39–5.57, *P* = .0001). Participants who had 2 sexual partners have you had in the past 2 years had a significant higher odd of having good knowledge (OR = 2.05, 95% CI = 1.02–4.12, *P* = .04), Table 5.

**Table 5:** Logistic regression analysis of demographic characteristics and knowledge level.

	Variables	OR (95% CI)	*P*-value
Marital status	Single	Reference	
	Married	0.77 (0.45–1.33)	.350
	Divorced	1.12 (0.39–3.28)	.829
	Widowed	0.60 (0.09–4.16)	.608
Age	18 to 29	Reference	
	30 to 39	1.03 (0.62–1.71)	.911
	40 **to** 49	0.70 (0.40–1.23)	.214
	50 to 59	0.72 (0.35–1.46)	.361
	60-	1.01 (0.21–4.92)	.987
Living area	West	Reference	
	East	2.19 (1.45–3.33)	.0001*
	North	1.63 (0.80–3.29)	.176
	South	0.97 (0.64–1.49)	.905
	Middle	1.18 (0.74–1.88)	.496
Education level	Primary	Reference	
	Middle	0.23 (0.04–1.37)	.106
	High	0.46 (0.12–1.73)	.251
	University	0.60 (0.16–2.16)	.431
Occupation	Not working	Reference	
	Medical	3.65 (2.39–5.57)	.0001*
	Other	0.76 (0.52–1.09)	.136
Comorbidities	Yes	0.60 (0.36–1.01)	.053
How many sexual partners have you had in the past 2 yr?	0	Reference	
	1	1.35 (0.85–2.16)	.209
	2	2.05 (1.02–4.12)	.045*
	3 or more	1.04 (0.51–2.12)	.921

OR = odds ratio.

## 4. Discussion

Regarding the transmission of HPV, a total of 452 participants correctly identified sexual contact as the main cause (34.6%), Prior research found that 54.8% of participating women reported that HPV is transmitted through sex and 53.5% declared that HPV is 1 of the reasons for cervical cancer in women.^[14]^ This indicates sufficient knowledge of the main causes of this virus in the community. Moreover, in this day and age, there is an increase in community awareness of sexually transmitted diseases, the implementation of multiple awareness campaigns, and the role of the media, schools, and universities have led to an increase in knowledge of this virus and its direct link to multiple sexual relationships and the diseases it can lead to. There has also been an increase in learning resources via the Internet, a focus on awareness campaigns for women, and an increase in talk about sexual health.

In this study, a total of 355 participants (43.3%) had a good knowledge score and 464 participants (56.7%) had poor knowledge. Prior research found that 69.2% of the participants have heard about HPV.^[14]^ In other studies, the results showed that increasing the vaccination rate has an impact on increasing the percentage of knowledge of HPV.^[15–17]^ According to another study in Brazil a total of 8581 participants, correctly identified HPV was 51.79% and 75.91% had an awareness of HPV vaccination. Women answered more questions correctly than men. Education level has an impact on the identification of HPV and vaccination as the low educational level was the variable that significantly influenced the level of knowledge about the virus and vaccination against it.^[18]^ 70% of young adults or more had awareness about HPV vaccination,^[18]^ 87% in the United States,^[19]^ and 75% in Italy.^[20]^ The results highlighted the vital role of educational programs about HPV and vaccination, so, depending on these results increasing awareness has a proportional relationship with the level of education so raising the level of consciousness by campaigning and emphasizing the role of education will lead to more control of this disease.

A total of 620 participants consulted a doctor if they noticed warts in the genital area 75.7%, and 78.8% advised their partner to receive the HPV vaccine. In comparison to another study, 26.4% participants women reported that genital warts could be the cause of HPV.^[21]^ Most of the participants (82.8%) advised those at high risk of contracting HPV to receive the vaccine. However, 81.7% advise women to have an annual Pap smear. In comparison to another study in the US between vaccinated women participants, 76.7% showed a history of HPV screening/Pap smear vs 87.80% in the unvaccinated group.^[21]^ According to the results of these studies the importance of knowing the symptoms of HPV, its causes, and how to prevent and treat is a main role in reducing the spreading of HPV and decreasing the main complications.

In this study, participants who lived in East area had significantly higher odds of knowledge compared to other areas (OR = 2.19, 95% CI = 1.45 − 3.33, *P* = .001). A prior study reported that the awareness was different in urban areas compared with rural ones. A total of 76.5% of women from urban areas were aware of HPV, while in rural areas, 60.8% heard about HPV.^[14]^ Based on these results, it is evident that there is a difference in the level of awareness depending on the geographical distribution, which plays a role in the level of awareness in these areas, according to that the importance of awareness campaigns has a vital role in reaching all areas.

When asked whether the vaccine protects against all types of HPV, only 275 participants (33.6%) correctly answered. According to another study conducted in Romania, 74.2% were confirmed about the vaccination practice in general (regardless the type of vaccine) and 283 participants 62.3% had heard about the HPV vaccine, 50.7% agreed with HPV vaccination.^[14]^ Furthermore, Regarding the desire to receive the HPV vaccine, the majority (n = 473,37.7%) have the desire, and 67 participants (8.2%) prevented to be vaccinated because they feel that they are not at risk. However, 55.8% of the participants showed that fear of side effects was the main barrier against HPV vaccination, and 13.2% because of no sex practice.^[14]^ Lack of awareness of the benefits of vaccines and unjustified fear of vaccines are concepts that need to emphasize the importance of community awareness to avoid the spread of the HPV.

In this study, most of the participants (62.1%) identified vaccination, early screening, and handwashing as the most effective methods to prevent infection. In terms of vaccination, 38.6% of participants confirmed its available in Saudi Arabia. prior study shows that vaccination is the key method of inhibition HPV. Moreover, using barriers like condoms and dental dams may decrease the diffusion of the virus.^[22]^ Regarding the recommended age for receiving the HPV vaccine, 37.7% correctly identified the 11 to 27 age range. In Romania, 68% of the women reported that they did not know the target population or the correct recommended ages for vaccination.^[14]^ Participants who had 2 sexual partners have you had in the past 2 years had a significantly higher odd of knowledge (OR = 2.05, 95% CI = 1.02–4.12, *P* = .04), Other studies have also noticed that 5.0% of the women who have not initiated sexual activity are clean with HPV or have a very low HPV incidence.^[23]^ All the previous results from numerous studies confirm the importance of supporting institutions and schools to spread awareness of these issues to reduce the risk of these diseases and the importance of early sexual awareness and methods of prevention and protection within organized and studied strategies to reach all groups.

Participants who worked in the medical field had significantly higher odds of knowledge compared to other jobs (OR = 3.65, 95% CI = 2.39–5.57, *P* = .0001). In another study conducted in the United States, medical and dental trainee students from 2 large academic centers were enrolled in an online pre-intervention questionnaire, followed by a 10-minute HPV educational, and then a post-intervention questionnaire, the correct answers increased from 28.4% (pre-intervention) to 51.9% (post-intervention; *P* < .01).^[24]^ Based on another study, it was noted that the information provided in school can be further disseminated through the media and health professionals to obtain more accurate answers, and combining the 2 methods has a major role.^[25]^ Discussing sexual health and HPV is very important and schools have a key role in raising awareness.^[26]^ Training of medical teams and students is necessary as well as changes in the strategies of education for the successful application of public health projects, boosting vaccination agreements, and strengthening the performance of screening tests.

## 5. Conclusion

This study identified that a considerable proportion of the study participants demonstrated poor level of knowledge of HPV vaccine. Participants who lived in Eastern area, those who worked in medical field, and those who had 2 sexual partners have you had in the past 2 years had a significant higher odd of having good knowledge. Future studies should be directed towards developing educational campaign to improve public awareness of HPV.

## Author contributions

**Conceptualization:** Mohammed Samannodi.

**Data curation:** Mohammed Samannodi.

**Formal analysis:** Mohammed Samannodi.

**Funding acquisition:** Mohammed Samannodi.

**Investigation:** Mohammed Samannodi, Shahad Mohammed Mousa, Lama Adnan Mandourah, Shayma Saleh Al-Rubaki, Maram Sulaiman Gubari, Wala Salem Fallatah, Sarah Tayeb, Mohammed A. Almatrafi, Mohammed Shaikhomer, Hassan Alwafi, Ahmad A. Imam, Enad Alsolami.

**Methodology:** Mohammed Samannodi.

**Project administration:** Mohammed Samannodi.

**Resources:** Mohammed Samannodi, Shahad Mohammed Mousa, Lama Adnan Mandourah, Shayma Saleh Al-Rubaki, Maram Sulaiman Gubari, Wala Salem Fallatah, Sarah Tayeb, Mohammed A. Almatrafi, Mohammed Shaikhomer, Hassan Alwafi, Ahmad A. Imam, Enad Alsolami.

**Software:** Mohammed Samannodi.

**Supervision:** Mohammed Samannodi.

**Validation:** Mohammed Samannodi.

**Visualization:** Mohammed Samannodi.

**Writing – original draft:** Mohammed Samannodi.

**Writing – review & editing:** Mohammed Samannodi, Shahad Mohammed Mousa, Lama Adnan Mandourah, Shayma Saleh Al-Rubaki, Maram Sulaiman Gubari, Wala Salem Fallatah, Sarah Tayeb, Mohammed A. Almatrafi, Mohammed Shaikhomer, Hassan Alwafi, Ahmad A. Imam, Enad Alsolami.
